# Examining the direction of association between attendance-related difficulties and maternal depression symptoms in two UK birth cohorts

**DOI:** 10.1007/s00737-026-01751-w

**Published:** 2026-09-22

**Authors:** Jennifer McGahan, Rebecca M Pearson, Holly Fraser, Amy Campbell, Jasmine Hearn, Elizabeth Braithwaite, Lucy Bowes, Jonathan Hill, Helen Sharp, Nicky Wright

**Affiliations:** 1 School of Psychology, Manchester Metropolitan University, Manchester, UK; 2 Bristol Medical School, University of Bristol, Bristol, UK; 3 Department of Experimental Psychology, University of Oxford, Oxford, UK; 4 Department of Primary Care and Mental Health, Institute of Population Health, University of Liverpool, Liverpool, UK

**Keywords:** Maternal mental health, School avoidance, School attendance, ALSPAC, WHCADS

## Abstract

**Purpose:**

Poor school attendance is a high priority for schools. Parental mental health is associated with school attendance problems, however, the direction of this relationship is unclear. This study examines the longitudinal direction of association between school attendance-related difficulties and parental mental health in two prospective UK birth cohorts.

**Methods:**

Data from two prospective birth cohorts were utilised: the Avon Longitudinal Study of Parents and Children (ALSPAC) (*n* = 6375) and the Wirral Child Health and Development Study (WCHADS) (*n* = 718).

**Results:**

In ALSPAC, school avoidant behaviours were associated with increased likelihood of subsequent maternal depression symptoms above clinical threshold (RR = 1.48 [95% CI 1.11, 2.00] *p* = 0.006) but maternal depression was not associated with later school avoidance (RR = 1.02 [95% CI 0.74, 1.40] *p* = 0.911). Similarly, separation anxiety behaviours were associated with increased maternal depression (RR = 1.20 [95% CI 1.21, 1.30], *p* < 0.001) but maternal depression did not predict later separation anxiety (RR = 1.06 [0.97,1.15] *p* = 0.237). In WCHADS, attendance-related difficulties at age 7 were associated with higher maternal depression symptoms at age 9 (b = 0.22, 95% CI 0.03, 0.42, *p* = 0.028), whereas maternal depression symptoms at age 7 were not associated with attendance-related difficulties at age 9 (OR = 0.98, 95% CI 0.78, 1.23, *p* = 0.869).

**Conclusions:**

We provide the first evidence that relationships between maternal mental health and school attendance-related difficulties most likely operate from child behaviours to maternal depression, not the other way around. This indicates that attendance interventions should replace parenting blaming narratives and instead integrate support for parents when issues arise.

**Supplementary Information:**

The online version contains supplementary material available at https://doi.org/10.1007/s00737-026-01751-w.

## Introduction

The national school attendance crisis in the UK is not improving (Children’s Commissioner for England, 2022) recent data found 1.8 million pupils in England were persistently absent from school (10% absence or more) (DfE, 2025). Understanding antecedents of poor school attendance is vital, as school absence negatively impacts life-long outcomes via employment, uptake of further education, peer relationships (Gonzálvez et al. 2019) and academic attainment (Gottfried 2014). However, to support effective interventions, there is a need for a more nuanced understanding of established risk factors including pupil anxiety and depression (Egger et al. 2003; Finning et al. 2019a), special educational needs and disabilities, disadvantaged backgrounds (Roberts and Long 2025) and poor family functioning (Kearney 2008).

Understanding how family factors cause and maintain attendance problems is particularly pertinent for the development of effective interventions. Studies show that effective school reintegration requires multi-level, ecologically situated interventions that consider facilitators and barriers and crucially prioritise parent/school collaboration (Corcoran et al. 2022; Kearney and Graczyk 2020). Parent mental health is a salient, yet poorly understood factor in school attendance; more attendance problems are consistently associated with higher rates of parental psychopathology (Chockalingam et al. 2023; Tekin and Aydın 2022; Egger et al. 2003). However, this is based on cross-sectional studies that report higher parent self-report of depression and anxiety is associated with more attendance problems (Bahali 2011; Bernstein and Garfinkel 1988; Carless et al. 2015; Özlem Ö, 2006). Despite requests for clarification of directionality in attendance and parent mental health (Carless et al. 2015; Chockalingam et al. 2023) and more general calls encouraging educational scientists to use longitudinal population datasets to enrich research (Cave and von Stumm 2021), and to our knowledge, these methods have not been used to explore directionality in school attendance problems and parental mental health.

Interestingly, the direction of this relationship has been presupposed; inferring that parents with mental health problems may have reduced capacity to monitor and support their child, leading to attendance problems (Ingul et al. 2019). However, it is entirely plausible that when a child avoids school, this presents significant challenges for parents and may lead to the onset of new parental depression and anxiety symptoms. Exploring directionality of attendance and parent mental health would build on previous work on bidirectional associations between child and parent depression (Wesseldijk et al. 2018) and anxiety (Silverman et al. 2021). Where the presumed causal dynamic (parent-to-child transmission) was deemed inadequate, the association was as much the result of child anxiety causing parent anxiety, as vice versa (Silverman et al. 2021). Similarly, recent work also suggests that parental psychopathology symptoms do not exert a direct, clinically meaningful effect on children’s academic achievement (Demange et al. 2026). Therefore, whilst parental mental health is clearly important, the direct effect on child educational outcomes may be less than presumed.

It is reasonable to propose that the impact of poor attendance on parent mental health may differ depending on the ‘type’ of attendance problem including school refusal, school phobia, truancy, separation anxiety (anxiety about being separated from a caregiver when attending school)(Elliott 1999; Heyne et al. 2004; Heyne et al., 2019) and (more recently) emotionally based school non-attendance. This range of ‘subtypes’ highlights a highly heterogeneous group, therefore it can be more helpful to consider the reasons ‘why’ pupils do not attend (Kearney 2007). For instance, pupils may be avoidant (e.g., fearful of a specific aspect of school, school is perceived as dangerous or dislikes socially evaluative situations) (Egger et al. 2003), engaging in more rewarding activities outside of school (Kearney 2007), anxious about leaving a caregiver (separation anxiety) (Finning et al. 2019b) or a combination of these factors.

Separation anxiety disorder is established as a strong predictor of school refusal, in both children and adolescents (Elliott., 1999; Tekin and Aydın 2022), to such an extent that earlier work used the term as a synonym for school attendance problems (e.g., school phobia/school refusal) (Hanna et al. 2006). Whilst separation anxiety is *one* explanation, it fails to consider important external variables such as fear of the school environment (Egger et al. 2003). Therefore, the current study must consider reasons ‘why’ pupils are absent separately, and how they may present distinct challenges for parent mental health. For instance, a child with an intense fear that harm will come to their caregiver when apart (separation anxiety) may impact parent mental health differently to a child who cannot attend because they find the school environment threatening. Additionally, a child with a depressed parent may worry about leaving them, in this case the parent mental health issue may precede a separation anxiety-based attendance issue.

Developing better methods for early identification is vital, as poor attendance in adolescence is considered more entrenched and difficult to address (Heyne et al. 2014). Despite much of the literature in this area focusing on adolescence, attendance problems identified from the first year of schooling can predict later issues (Hancock et al. 2018) as mild or emerging issues commonly start in childhood rather than adolescence (mean age 10.9 years) (Egger et al. 2003). There is currently a lack of research exploring how specific family factors, such as parent mental health, are associated with the emergence of attendance issues (Ingul et al. 2019), especially pre-adolescence (separation anxiety Vs disliking the school environment). Understanding how early attendance issues correlate with parental mental health could inform much needed preventative support.

Dominant educational policies are currently reactive and typically wait for the pupil to reach a threshold of ‘persistent absence’ before a plan to improve attendance commences. In the UK, schools emphasise the legal responsibility of parents to provide their child with a full-time education, echoed in updated school safeguarding guidance that now includes ‘educational neglect’ when a parent/carer fails to ensure a child receives an education, including persistent school absences (DfE, 2024a). This deficit narrative places blame and stigma on parents and, if schools perceive parental non-compliance during this process, it can escalate to education supervision and school attendance orders, school absence fines and prosecution (DfE, 2024b). These policies diminish the complexity of addressing attendance problems and heighten parent stress at an already challenging time for families; potentially impacting parent mental health.

### Aim of the current studies

We examined the association between school attendance-related difficulties and parent mental health via two longitudinal datasets using an epidemiology cohort study design. Study one examined associations between both separation anxiety (specifically in the context of school) and school avoidant behaviours in children aged 8 years, using child and parent report, and maternal depression symptom scores above clinical cut off before (child aged 6 years) and after (child aged 11 years). Study 2 used a second UK cohort and a broader indicator of attendance-related difficulties measured at two time points, alongside maternal depression (at ages 7 and 9 years), enabling a formal test of reciprocal longitudinal association. Thus, we advance existing knowledge on this topic by robustly testing directionality of effects. We hypothesised a bidirectional relationship between school attendance-related difficulties and parental mental health symptoms.

### Study 1: Materials and methods

#### Participants

The sample comprised participants from the Avon Longitudinal Study of Parents and Children (ALSPAC) (Boyd et al. 2013; Fraser et al. 2013; Northstone et al. 2019) an ongoing population-based birth cohort beginning in pregnancy in 1991. Detailed information for the cohort, including ethical information and informed consent procedures, is provided in the Supplementary Information. Please note that the study website contains details of all the data that is available through a fully searchable data dictionary and variable search tool” and reference the following webpage: http://www.bristol.ac.uk/alspac/researchers/our-data/. The ALSPAC cohort is broadly considered representative of the wider population in England despite fewer participants from lower SES and minority ethnic backgrounds (Boyd et al. 2013).

#### Measures

Maternal depression symptoms were measured via the Edinburgh Postnatal Depression Scale (EPDS) (Cox et al. 1987). The EPDS is an extensively used measure that was originally developed to measure depression in the perinatal period using 10-items to assess symptoms over the previous week. It is now routinely used outside of the perinatal period (Paul and Pearson 2020). The EPDS has been administered at 11 timepoints with ALSPAC mothers and in the current study we use data when the EPDS was completed at child ages 6, 8 and 11 years. See Supplementary materials for a description of how patterns were derived for the EPDS depression scores.

#### School attendance behaviours

Two proxy measures for school attendance-related difficulties were generated from parent and child completed school questionnaires, administered when the child turned 8 years and 1 month (Table 1). The two proxy measures related to (1) avoidant school behaviour (staying off school when not unwell/on holiday) and (2) school specific separation anxiety behaviours (school induces anxiety due to being away from the caregiver). Combining several questionnaire items to generate proxy measures is an established approach when working with population-based longitudinal cohorts (Nilsson et al. 2023). Precedent for a proxy measure of school attendance behaviours comes from another longitudinal study; the Great Smokey Mountains Study, where the ‘anxious school refusers’ proxy combined four questions on worry-related attendance (parent and child responses) (Egger et al. 2003). As discussed above, despite their proxy measure of anxious school refusers indicating elevated rates of separation anxiety (5–18%) the two were clearly not synonymous, with fear of school being a more common fear. For this reason, we created two separate proxy measures using available ALSPAC questions: separation anxiety and avoidant see Table 1.

**Table 1:** Individual ALSPAC To C6c questions used to develop the proxy measures of school attendance problems (avoidant/ anxious). See supplementary materials for more information including sample

Question / item	Respondent	ALSPAC variable name	Proxy measure	*N* response per question
“Frequency child likes going to school at 8 years old”	Child	ccc100	Avoidant	7625
“Frequency child feels happy at school”	Child	ccc170	Avoidant	7648
“Number of times child has stayed away from school when not sick except for holidays”	Child	ccc280	Avoidant	7604
“Frequency child looks forward to going to school”	Parent	kr620	Avoidant	8194
C6c: Degree to which child’s separation anxieties interfered with school/learning	Parent	Kr219	Anxious	525
C3c: Degree to which child was unwilling to go to school in case something nasty happened to special people in past month relative to peers	Parent	Kr203	Anxious	7599

#### Analysis

Multinomial logistic regression was used to measure the association between school attendance measures (exposure) and presence of high depression. Defined as above 12 points on the EPDS across the 2 time points as a categorical outcome variable. Relative Risk Ratios (RRRs) were computed using ‘asymptomatic at either time point’ as the baseline comparison. Unadjusted logistic regression models were computed, followed by models adjusted for maternal education and child sex. Analysis was undertaken in Stata version 18 (StataCorp 2023).

#### Results

Boys and girls showed similar school avoidant behaviour scores (males mean = 2.14, SD = 0.28, females mean = 2.09, SD = 0.26) and separation anxiety behaviour scores (males mean = 0.72, SD = 0.43, females mean = 7.15, SD = 0.39). The correlation between school avoidant and separation anxiety scores was 0.46 *p* < 0.001. Regarding links with maternal depression symptoms, in unadjusted models, for both school attendance outcomes there was evidence for associations between attendance behaviours and later maternal depression symptoms above cut off, but not earlier maternal depression. As shown in Table 2, avoidant school behaviour was associated with increased maternal depression after (RR = 1.54 [95% CI 1.17 to 2.02) *p*=0.002) but not before (RR = 1.07 [95%CI 0.79 to 1.44] *p* = 0.657) with a similar pattern for school separation anxiety (depression after RR = 1.2 [95% CI 1.1 to 1.3] *p* < 0.001; depression before RR = 1.1[95% CI 0.9 to 1.2] *p* = 0.124). The results were similar after adjustment for maternal age, maternal education, income level, marital status, number of children and child sex (school avoidance behaviour: depression after RR = 1.5 [95% CI 1.11 to 2.00] *p* = 0.006, depression before RR = 1.0 [95% CI 0.74 to 1.40] *p* = 0.911; school separation anxiety behaviour: depression after RR = 1.26 [95% CI 1.212 to 12.300] *p* < 0.001, depression before RR = 1.1 [95% CI 0.96 to 1.15] *p* = 0.237). The group of mothers who were above the cut off for depression at both time points was small, and results suggested no significant associations with maternal depression before or after attendance behaviours.

**Table 2:** Multinomial Logistic Regression Results: Association between school attendance problems and maternal depression using ALSPAC data

Depression Status	RR for a SD increase in total School Anxiety subscale, 95%CI, *p*	RR for a SD increase in total School Anxiety subscale, CI, *p* (adjusted for maternal age, maternal education, income level, marital status, number of children and child sex)	RR for a SD increase in total Avoidant School Behaviours Subscale, CI, *p*	RR for a SD increase in total School Avoidant Behaviours Subscale, CI, *p* (adjusted for maternal age, maternal education, income level, marital status, number of children and child sex.)
Not depressed either time (*n* = 3873 70%)	REF (so set to 1)		REF (so set to 1)	
Depressed (> 12 on EPDS) only at age 6 (before School probs)(*n* = 663, 12%)	1.07 (1.00 to 1.16) *p* = 0.124	1.06 (0.97 to 1.15) *p* = 0.237	1.07 (0.79 to 1.44) *p* = 0.657	1.02 (0.74 to 1.40) *p* = 0.911
Depressed only at age 10 (after school probs) (*n* = 883, 16%)	1.22 (1.14 to 1.31) *p* < 0.001	1.20 (1.12 to 1.29) *p* < 0.001	1.54 (1.17 to 2.02) *p* = 0.002	1.48 (1.11 to 2.00) *p* = 0.006
Depressed at both age 6 and 10 (*n* = 96, 2%.)	1.13 (0.93 to 1.37) *p* = 0.203	1.11 (0.91 to 1.36) *p* = 0.306	1.54 (0.72 to 3.33) *p* = 0.265	1.44 (0.66 to 3.17) *p* = 0.360

### Study 2: materials and methods

This study examined reciprocal longitudinal associations between maternal depression and school attendance-related difficulties (reported by parent and teacher) with all data available at two timepoints; child aged 7 and 9 years.

#### Participants

The sample comprised participants from the Wirral Child Health and Development Study (WCHADS) a prospective epidemiological study starting in pregnancy in 2006. WCHADS is based in a different region of the UK with the sample born nearly two decades apart from ALSPAC, allowing replication of study 1 across a different geographical and historical context. Detailed information for the cohort, including ethical information and informed consent procedures, is provided in the Supplementary Information.

#### Measures

Maternal depression symptoms were assessed using the 20-item Centre for Epidemiological Studies – Depression (CES-D) (Radloff 1977) at child age 7 and the 9-item Patient Health Questionnaire-9 (PHQ-9) at child age 9 (Kroenke et al. 2001). As different instruments were used across the two waves, scores were standardised within wave (mean = 0.00, SD = 1.00) prior to analysis to facilitate comparability. Higher scores reflect greater depression symptoms.

Items from existing measures were selected to assess child attendance-related difficulties, unlike ALSPAC, the available WCHADS items did not permit differentiation between avoidance-related and separation anxiety-related attendance difficulties. Consequently, a broader attendance-related difficulties indicator was derived via available items that assessed fears about attending school, truancy/skipping school, and requests to leave or return home from school (see Table 3). As the children were aged 7 and 9 years, the truancy question was included to reflect days off school when they should be present, as in a UK context children are expected to be taken to school by a caregiver and therefore to be away from school without a caregivers knowledge would be highly unlikely. For more information on this proxy measure and description of the covariates included, see supplementary materials.

**Table 3:** Items used to create school attendance problems variables in WCHADS

Reporter	Item	Age 7 *N* (%)	Age 9 *N* (%)
Mother	Fears going to school		
	*Not true*	678 (94.56%)	669 (93.31%)
	*Sometimes true*	33 (4.60%)	37 (5.16%)
	*Very true*	6 (0.84%)	11 (1.53%)
Teacher	Fears going to school		
	*Not true*	662 (97.21%)	658 (96.62%)
	*Sometimes true*	17 (2.50%)	22 (3.23%)
	*Very true*	2 (0.29%)	1 (0.15%)
Mother	Truancy, skips school		
	*Not true*	717 (100%)	717 (99.86%)
	*Sometimes true*	0	1 (0.14%)
	*Very true*	0	0
Teacher	Truancy, skips school		
	*Not true*	670 (98.38%)	674 (98.97%)
	*Sometimes true*	8 (1.17%)	7 (1.03%)
	*Very true*	3 (0.44%)	0
Teacher	This child makes up reasons to go home from school		
	*Doesn’t apply*	627 (0.29%)	639 (92.74%)
	*Sometimes applies*	50 (7.36%)	47 (6.82%)
	*Certainly applies*	2 (0.29%)	3 (0.44%)

#### Analysis

We estimated two-wave cross-lagged panel models to examine reciprocal associations between child attendance-related difficulties at age 7 and maternal depression symptoms at age 9, while accounting for the stability of each construct over time. Specifically, the models assessed both the prospective association between attendance-related difficulties at age 7 and maternal depression symptoms at age 9, and the reverse pathway from maternal depression symptoms at age 7 to attendance-related difficulties at age 9.

Models were estimated in Stata version 19.5 (StataCorp 2025)using generalised structural equation modelling (GSEM). Maternal depression symptoms were modelled using a Gaussian distribution with an identity link function, whereas attendance-related difficulties were modelled using a Bernoulli distribution with a logit link function. Robust standard errors were used throughout, and parameters were estimated using maximum likelihood. An initial model included only the autoregressive and cross-lagged pathways. A second model additionally adjusted for maternal age at pregnancy, maternal education, marital/cohabitation status, family deprivation and child sex. Residual covariances were not specified because residual covariances between Gaussian and binary outcomes are not identified within the GSEM framework. To aid interpretation, maternal depression symptoms were standardised within wave; coefficients from the maternal depression equation therefore represent differences in depression symptoms in standard deviation units. Coefficients from the attendance equation are reported as odds ratios (ORs).

#### Results

School attendance-related difficulties were reported for 13.37% (*N* = 96) of children at age 7 years and 15.06% (*N* = 108) at age 9 years. There was no difference in rates for boys versus girls (11.72% versus 15.52%, *p*=0.125, at age 7, 14.16% versus 15.90%, *p*=0.510, at age 9).

Results from the cross-lagged panel models are presented in Table 4. In the initial model, maternal depression symptoms demonstrated substantial stability between ages 7 and 9 (b = 0.53, *p* < 0.001), and school attendance-related difficulties also showed strong continuity over time (OR = 4.11, 95% CI 2.53–6.63, *p* < 0.001). Attendance-related difficulties at age 7 were associated with higher maternal depression symptoms at age 9 (b = 0.26, 95% CI 0.06–0.45, *p* = 0.013). In contrast, maternal depression symptoms at age 7 were not associated with subsequent attendance-related difficulties at age 9 (OR = 1.07, 95% CI 0.87–1.32, *p* = 0.532).

**Table 4:** Cross-lagged associations between child attendance problems (age 7) and maternal depressive symptoms (age 9) WCHADS

Predictor (Age 7)	Unadjusted model		Adjusted model	
	b (SE) / OR (95% CI)	*p*	b (SE) / OR (95% CI)	*p*
Outcome: Maternal depression symptoms (Age 9)				
Maternal depression symptoms	0.53 (0.05)	< 0.001	0.51 (0.05)	< 0.001
Attendance problems	0.26 (0.10)	0.013	0.22 (0.10)	0.028
Maternal age at pregnancy	–	–	−0.01 (0.01)	0.060
Degree or higher education	–	–	−0.12 (0.06)	0.045
Female child	–	–	0.05 (0.06)	0.382
Married/cohabiting	–	–	−0.17 (0.11)	0.104
Outcome: Attendance problems (Age 9)				
Maternal depression symptoms	1.07 (0.87–1.32)	0.532	0.98 (0.78–1.23)	0.869
Attendance problems	4.11 (2.53–6.63)	< 0.001	3.88 (2.34–6.40)	< 0.001
Maternal age at pregnancy	–	–	0.99 (0.95–1.03)	0.672
Degree or higher education	–	–	0.54 (0.32–0.93)	0.026
Female child	–	–	1.21 (0.79–1.86)	0.390
Married/cohabiting	–	–	0.54 (0.31–0.92)	0.024

Note. Maternal depressione symptoms were standardised within wave. Coefficients are unstandardised betas for the maternal depression equation and odds ratios (ORs) for the attendance equation

Adjustment for maternal age at pregnancy, maternal education, marital/cohabitation status, family deprivation and child sex had little impact on the pattern of findings. Maternal depression symptoms remained stable across time (b = 0.51, *p* < 0.001), and attendance-related difficulties at age 7 continued to predict higher maternal depression symptoms at age 9 (b = 0.22, 95% CI 0.02–0.41, *p* = 0.028). Attendance-related difficulties also remained strongly stable over time (OR = 3.88, 95% CI 2.34–6.40, *p* < 0.001). Consistent with the initial model, maternal depression symptoms at age 7 did not predict later school attendance-related difficulties (OR = 0.98, 95% CI 0.78–1.23, *p* = 0.869). To examine whether findings could be explained by shared-informant effects, a sensitivity analysis was conducted using teacher-reported attendance-related difficulties only. Findings were substantively unchanged (Supplementary Table S1). Teacher-reported attendance-related difficulties at age 7 remained associated with higher maternal depression symptoms at age 9 (b = 0.26, 95% CI 0.02–0.49, *p* = 0.031), whereas maternal depression symptoms at age 7 were not associated with subsequent attendance-related difficulties (OR = 0.99, 95% CI 0.79–1.24, *p* = 0.932).

## Discussion

This study provides the first longitudinal evidence, replicated across two UK birth cohorts, that relationships between maternal mental health and school attendance-related difficulties most likely operate from child behaviours to maternal depression, not the other way around. Across both cohorts, children experiencing attendance difficulties were more likely to have mothers who subsequently reported higher levels of depression symptoms, whereas there was little evidence that maternal depression symptoms predicted the later emergence of attendance-related difficulties. The consistency of findings across cohorts recruited from different regions of the UK born nearly two decades apart strengthens confidence in this temporal pattern.

The relationship between poor parental mental health and attendance-related difficulties required clarification, particularly in terms of how attendance difficulties impact parent mental health (Carless et al. 2015; Chockalingam et al. 2023). The current study advances existing knowledge by challenging the common assumption that parent mental health is primarily a cause of children’s attendance problems. In Study 1 we examined separation anxiety and avoidance separately and found that both ‘types’ of attendance avoidance behaviours contributed similarly to the development of new cases of parental depression, despite these representing conceptually distinct pathways. Although separation anxiety reflects anxiety about being separated from a caregiver, whereas school avoidance is commonly driven by fears relating to the school environment itself (Egger et al. 2003; Elliott 1999; Heyne et al. 2004; Heyne et al., 2019) both appeared to have similar implications for subsequent maternal mental health. This suggests it may be the broader experience of managing persistent attendance-related difficulties, rather than the specific reason underlying them, that contributes to poorer maternal mental health.

The finding extends existing work exploring how child psychopathology contributes to parent depression (Johnco et al. 2021; Murray et al. 2009) and responds to a specific call for a better understanding of ‘child factors’ and parent psychopathology (England and Sim 2009). Our results are consistent with the hypothesis that the sustained (often daily) parenting challenges of attendance difficulties(Hanna et al. 2006) and associated disruption to daily life (missing work, childcare issues, losing employment) can lead to the development of maternal depression (Marchbanks et al. 2015). These findings highlight a need for early, non-judgmental parental support; partnering with parents and viewing them as the ‘expert’ in their child may reduce the additional burden of perceived blame. The cumulative impact of managing attendance difficulties alongside potentially shame-inducing educational policies (fines and parent contracts) is currently unclear and warrants further investigation. Future research should explore protective and risk factors for mental health issues in parents of school-avoidant children, including systemic factors (individual school, local authority, government) as well as individual level factors known to correlate with attendance issues such as parental self-efficacy(Carless et al. 2015) and the broader context of family functioning (Kearney 2022).

Considering this work, the current approach to improving attendance in the UK (including education supervision and school attendance orders, school absence fines and prosecution)(DfE, 2024b) clearly has the potential to exacerbate, rather than alleviate parental mental health issues. Similar punitive approaches are adopted internationally despite limited evidence of their effectiveness; the stubbornly low attendance rates in the UK attest to this. These approaches are considered ineffective because they are based on the problematic assumption that most attendance problems are the result of parent motivation, and consequently overlook other risk factors (Hancock et al. 2018). Our study supports this; failure to consider parental mental health issues when devising approaches such as attendance plans and parenting contracts, is a barrier to improving attendance. It is important that schools and local authorities understand the substantial impact school avoidant behaviours can have on parental mental health and adapt approaches accordingly.

Strengths & limitations:

A key strength of the study was the use of two prospective longitudinal cohort studies with repeated assessments of maternal depression symptoms, facilitating examination of the temporal ordering of associations between maternal mental health and child school attendance behaviours. The inclusion of two independent cohorts recruited from different regions of the UK and born nearly two decades apart enabled replication of findings across distinct developmental, geographical and historical contexts. The broadly similar pattern of findings across cohorts, despite differences in study design and measurement, strengthens confidence that the observed associations are not specific to a single sample or period. In addition, separating different manifestations of attendance difficulties in ALSPAC (avoidance and separation anxiety) provided a more nuanced understanding of the relationship between maternal depression symptoms and school attendance problems. Finally, the availability of both parent and teacher reports within WCHADS enables sensitivity analyses that reduced concerns regarding shared-informant effects, with findings remaining substantively unchanged when attendance behaviours were derived solely from teacher reports.

Several limitations should also be considered. First, both studies relied on proxy indicators of school attendance difficulties derived from questionnaire items rather than measures designed specifically to assess school attendance problems. Whilst combining multiple items to derive constructs is common practice in longitudinal cohort studies, future research would benefit from using validated measures that distinguish between different forms and functions of attendance difficulties and are co-produced with schools, families and young people. Relatedly, neither study had access to objective school attendance records. Consequently, the measures used here capture behaviours and difficulties associated with attendance problems rather than actual absence from school.

Second, the WCHADS attendance items were infrequently endorsed, resulting in highly skewed distributions and limited variability. To address this, a binary indicator reflecting the presence of any attendance difficulty was created. Whilst this approach maximised statistical power and reduced problems associated with sparse data, it inevitably reduced information regarding severity, frequency and type of attendance difficulty. The resulting estimates may therefore represent conservative estimates of the true association between attendance problems and maternal mental health. In contrast to ALSPAC, the available WCHADS items did not permit separation of avoidance and separation anxiety-related attendance difficulties, limiting direct comparison of attendance subtypes across cohorts. Third, different measures of maternal depression symptoms were used across timepoints in WCHADS (CES-D at age 7 and PHQ-9 at age 9). Scores were standardised within wave prior to analysis to facilitate longitudinal modelling. Although both instruments are widely used and well-validated measures of depression symptoms, differences in scale content and structure may have introduced additional measurement variability.

Fourth, both cohorts were recruited from specific regions of the UK and contained relatively low ethnic diversity. The ALSPAC cohort lacks under-represented families from lower socioeconomic backgrounds. In addition, attrition in both cohorts resulted in analytic samples that were somewhat more socioeconomically advantaged than the original recruited samples. Consequently, caution is warranted when generalising findings to more ethnically diverse populations or to areas experiencing different socioeconomic conditions. Finally, we focused exclusively on maternal depression symptoms and were unable to examine paternal mental health. To fully understand the role of parental mental health in the development and maintenance of school attendance difficulties, future studies should consider mental health across multiple caregivers and wider family systems.

## Conclusion

In conclusion, we did not find a bidirectional relationship between maternal depression symptoms and school attendance-related difficulties. School attendance-related difficulties were associated with maternal depression only after the occurrence of child attendance problems, not before. We provide evidence that school attendance-related difficulties increase the likelihood of mothers’ later depression symptoms. This evidence highlights the importance of developing non-judgemental, early support for parents; failing to consider this may be a barrier to effective educational policy and outcomes.

## Supplementary Information

Below is the link to the electronic supplementary material.

Supplementary Material 1 (DOCX 79.9 KB)

## Data Availability

Data availability statement: The data analysed in this study is subject to the following licenses/restrictions: The steps below highlight how to apply for access to the data included in the data note and all other ALSPAC data: (1) Please read the ALSPAC access policy (http://www.bristol.ac.uk/media-library/sites/alspac/documents/researchers/data-access/ALSPAC_Access_Policy.pdf) which describes the process of accessing the data and samples in detail, and outlines the costs associated with doing so. (2) You may also find it useful to browse our fully searchable research proposals database (https://proposals.epi.bristol.ac.uk/?q=proposalSummaries), which lists all research projects that have been approved since April 2011. (3) Please submit your research proposal (https://proposals.epi.bristol.ac.uk/) for consideration by the ALSPAC Executive Committee. You will receive a response within 10 working days to advise you whether your proposal has been approved. Requests to access these datasets should be directed to https://proposals.epi.bristol.ac.uk/.WCHADS data: Due to ethical constraints the WCHADS data cannot be made openly available. Supporting data are available to bona fide researchers on approval of an application for access. Further information about the data and conditions for access are available at the University of Liverpool Research Data Catalogue: https://doi.org/10.17638/datacat.liverpool.ac.uk/564.

## Declarations

### Clinical trial number

: not applicable.
