# Supplementary material for: Examining the direction of association between attendance-related difficulties and maternal depression symptoms in two UK birth cohorts
Source: Arch Womens Ment Health. 2026 Sep 22;29(5):131. doi: 10.1007/s00737-026-01751-w (PMC13597554; doi:10.1007/s00737-026-01751-w)
Supplement: Supplementary file 1 [file 737_2026_1751_MOESM1_ESM.docx]

# Supplementary information

### ALSPAC:

ALSPAC is a longitudinal pregnancy cohort which aimed to recruit all pregnant women in the former county of Avon with an expected due date between April 1991 and December 1992. Detailed information has continued to be collected on mothers, partners and children in the cohort, this process has been described in detail elsewhere (Boyd et al., 2013; Fraser et al., 2013; Northstone et al., 2019). Ethical approval for the study was obtained from the ALSPAC Ethics and Law Committee and the Local Research Ethics Committees. Informed consent for the use of data collected via questionnaires and clinics was obtained from participants following the recommendations of the ALSPAC Ethics and Law Committee at the time. Please note that the study website contains details of the data that is available through a fully searchable data dictionary and variable search tool <https://variables.alspac.bris.ac.uk/>.

Pregnant women resident in Avon, UK with expected dates of delivery between 1st April 1991 and 31st December 1992 were invited to take part in the study. 20,248 pregnancies have been identified as being eligible and the initial number of pregnancies enrolled was 14,541. Of the initial pregnancies, there was a total of 14,676 foetuses, resulting in 14,062 live births and 13,988 children who were alive at 1 year of age. When the oldest children were approximately 7 years of age, an attempt was made to bolster the initial sample with eligible cases who had failed to join the study originally. As a result, when considering variables collected from the age of seven onwards (and potentially abstracted from obstetric notes) there are data available for more than the 14,541 pregnancies mentioned above. The number of new pregnancies not in the initial sample (known as Phase I enrolment) that are currently represented in the released data and reflecting enrolment status at the age of 24 is 906, resulting in an additional 913 children being enrolled (456, 262 and 195 recruited during Phases II, III and IV respectively). The phases of enrolment are described in more detail in the cohort profile paper and its update (Boyd et al., 2013; Fraser et al., 2013). The total sample size for analyses using any data collected after the age of seven is therefore 15,447 pregnancies, resulting in 15,658 foetuses. Of these 14,901 children were alive at 1 year of age. Of the original 14,541 initial pregnancies, 338 were from a woman who had already enrolled with a previous pregnancy, meaning 14,203 unique mothers were initially enrolled in the study. As a result of the additional phases of recruitment, a further 630 women who did not enrol originally have provided data since their child was 7 years of age.

This provides a total of 14,833 unique women enrolled in ALSPAC as of September 2021. The present study focuses on 5,515 mothers and children who had complete data on the measures used in this analysis.

### Measures

We derived EPDS patterns for mothers to measure the change in EPDS symptoms over the course of approximately 5 years, taking data from when the child was 6 years and when the child was 11 years. Patterns were derived for the EPDS depression scores. Being symptomatic was taken as having a score above 12 on the EPDS (Cox et al., 1987; Murray & Carothers, 1990) following previous ALSPAC publications (Paul & Pearson, 2020). These groups were coded as ‘0 = Asymptomatic at both timepoints’; ‘1 = Symptomatic at age 6 only’; ‘2= Symptomatic at age 11 only’; and ‘3 = Symptomatic at both timepoints’. Numbers in each category are given in table 2. Descriptive data relating to the EPDS data in ALSPAC has been published previously (Paul & Pearson, 2020). To see the number of mothers in each group see table 2.

The individual items used for each school avoidance behaviour proxy measure are available in table 1. The parent questionnaire was sent out to 11251; there were 8269 recorded responses. This is 73.5% of the questionnaires sent out. The ALSPAC parent questionnaire ‘My Son / Daughter’s Well-being’ utilised the parent version of the Development and Well-being Assessment (DAWBA) (Goodman et al., 2000). We used individual items (KR620/KR219/KR203) to align with our research question. The response rates for each of these questions are included in table 1. Of note, question KR219 has much fewer responses (*n-*525) than the other questions from the parent questionnaire. This is because *only* parents who responded ‘a lot more than others’ to questions on separation anxiety (the preceding section of the questionnaire) completed this question. The child questionnaire was sent out to 11,147 children; there were 7,417. This is 67% of the questionnaires sent out. Child report items were taken from the ALSPAC child questionnaire ‘Me and My School’. We included individual items (CCC100, CCC170, CCC280), the response rate for each is included in table 1.

### WCHADS

The Wirral Child Health and Development Study (WCHADS) is a prospective epidemiological study starting in pregnancy Ethical approval was granted by the Cheshire North and West Research Ethics committee and all women gave written informed consent at the point of recruitment in the antenatal clinic and at every assessment point. Parents gave consent for schools to be contacted to collect teacher questionnaires.

The cohort comprised 1,233 first-time expectant mothers recruited in pregnancy with a live, singleton baby for long-term follow-up post-birth (see Sharp et al., 2012for a detailed account of sampling). The participants were identified from consecutive first-time mothers who booked for antenatal care at 12 weeks’ gestation between 12/02/2007 and 29/10/2008. The booking clinic was administered by the Wirral University Teaching Hospital, which was the sole provider of universal prenatal care on the Wirral Peninsula. Socioeconomic conditions on the Wirral range between the deprived inner city and affluent suburbs, but with low numbers from ethnic minorities. The mean age at recruitment was 26.8 years (*SD* = 5.8, range 18–51), 41.8% of the sample were in the most deprived quintile of UK neighbourhoods (Noble et al., 2004) and 96.1% were White British. The study has collected 13 waves of data from 20 weeks gestation up to child age 13 years. At age 7 years, 776 mothers and 728 teachers, and at age 9 751 mothers and 742 teachers completed questionnaires.

### Participants

Our analyses focused on 718 participants who provided data at age 7 (mean age 7.35 [SD = .31] years) and 9 years (mean age 9.45 [SD = .42] years). The sample comprised a similar number of female (52.65%, N=340) and male children (47.35%, N=378), the mean mother age at first pregnancy was 27.93 (SD = 5.63), 33.57% (N=241) of mothers had a university degree or higher, 81.75% (N=587) of mothers were married or cohabiting when pregnant with study child and 36.92% (N=264) of families were living in the most deprived quintile of UK neighbourhoods. Compared to the originally recruited 1233 families, families where complete data was provided had a significantly older mean maternal age (p<.001), mothers’ were more likely to have a degree or higher education (p<.001), more likely to be married or cohabitating (p<.001) and less likely to be living in the most deprived quintile of UK neighbourhoods (p<.001).

### Attendance proxy measure

Two items were selected from mother report on the Child Behaviour Checklist and the same two items from teacher report on the Teacher Report Form (Achenbach & Rescorla (2000) the items are rated on a 3-point scale: 0 = not or never true, 1 = somewhat or sometimes true and 2= very or frequently true. One item was selected from the teacher reported MacArthur Health and Behaviour Questionnaire “child’s school experiences” subscale (Essex et al., 2002). The items and their endorsement rates are shown in Table 3. Due to the low prevalence and highly skewed distribution of the items, a single binary indicator reflecting the presence of any attendance difficulties (items rated as 1 or 2) based on parent and teacher reports was created for each age.

### Covariates

Covariates were measured at recruitment in pregnancy (and at birth for child sex). These included child sex at birth (coded as 0=male, 1 = female), maternal age in years at first pregnancy, maternal education (degree or higher vs not), maternal marital/cohabitation status (married/cohabiting versus not married/cohabiting) and family deprivation. Deprivation was measured using the revised English Index of Multiple Deprivation (IMD)^24^ based on data collected from the UK Census in 2001. According to this system, postcode areas in England are ranked from most deprived (i.e. IMD of 1) to least deprived (i.e. IMD of 32,482) based on deprivation in seven domains: income, employment, health, education and training, barriers to housing and services, living environment, and crime. All mothers were given IMD ranks according to the postcode of the area where they lived and assigned to a quintile based on the UK distribution of deprivation; a binary variable reflecting 1=most deprived and 0=all other quintiles, was used for analysis.

**References:**

Achenbach, T. M., & Rescorla, L. A. (2000). Manual for the ASEBA Preschool Forms & Profiles. *Burlington, VT: University of Vermont, Research Center for Children, Youth, & Families.*, *30*. https://www.scirp.org/reference/referencespapers?referenceid=1855203

Boyd, A., Golding, J., Macleod, J., Lawlor, D. A., Fraser, A., Henderson, J., Molloy, L., Ness, A., Ring, S., & Smith, G. D. (2013). Cohort Profile: the ’children of the 90s’--the index offspring of the Avon Longitudinal Study of Parents and Children. *International Journal of Epidemiology*, *42*(1), 111–127. https://doi.org/10.1093/IJE/DYS064

Cox, J. L., Holden, J. M., & Sagovsky, R. (1987). Detection of postnatal depression. Development of the 10-item Edinburgh Postnatal Depression Scale. *The British Journal of Psychiatry : The Journal of Mental Science*, *150*(JUNE), 782–786. https://doi.org/10.1192/BJP.150.6.782

Essex, M. J., Boyce, W. T., Goldstein, L. H., Armstrong, J. M., Kraemer, H. C., & Kupfer, D. J. (2002). The Confluence of Mental, Physical, Social, and Academic Difficulties in Middle Childhood. II: Developing the MacArthur Health and Behavior Questionnaire. *Journal of the American Academy of Child & Adolescent Psychiatry*, *41*(5), 588–603. https://doi.org/10.1097/00004583-200205000-00017

Fraser, A., Macdonald-wallis, C., Tilling, K., Boyd, A., Golding, J., Davey smith, G., Henderson, J., Macleod, J., Molloy, L., Ness, A., Ring, S., Nelson, S. M., & Lawlor, D. A. (2013). Cohort Profile: the Avon Longitudinal Study of Parents and Children: ALSPAC mothers cohort. *International Journal of Epidemiology*, *42*(1), 97–110. https://doi.org/10.1093/IJE/DYS066

Goodman, R., Ford, T., Richards, H., Gatward, R., & Meltzer, H. (2000). The Development and Well-Being Assessment: Description and Initial Validation of an Integrated Assessment of Child and Adolescent Psychopathology. *Journal of Child Psychology and Psychiatry*, *41*(5), 645–655. https://doi.org/10.1111/J.1469-7610.2000.TB02345.X

Murray, L., & Carothers, A. D. (1990). The validation of the Edinburgh Post-natal Depression Scale on a community sample. *The British Journal of Psychiatry : The Journal of Mental Science*, *157*(AUG.), 288–290. https://doi.org/10.1192/BJP.157.2.288

Noble, M., Wright, G., Dibben, C., Smith, G., McLennan, D., Anttila, C., Barnes, H., Mokhtar, C., Noble, S., & Gardner, J. (2004). The English indices of deprivation 2004. *London: Office of the Deputy Prime Minister*, *180*. https://www.research.ed.ac.uk/en/publications/the-english-indices-of-deprivation-2004/

Northstone, K., Lewcock, M., Groom, A., Boyd, A., Macleod, J., Timpson, N., & Wells, N. (2019). The Avon Longitudinal Study of Parents and Children (ALSPAC): an update on the enrolled sample of index children in 2019. *Wellcome Open Research*, *4*. https://doi.org/10.12688/WELLCOMEOPENRES.15132.1

Paul, E., & Pearson, R. M. (2020). Depressive symptoms measured using the Edinburgh Postnatal Depression Scale in mothers and partners in the ALSPAC Study: A data note. *Wellcome Open Research*, *5*, 108. https://doi.org/10.12688/WELLCOMEOPENRES.15925.2

Sharp, H., Pickles, A., Meaney, M., Marshall, K., & Tibu, F. (2012). Frequency of Infant Stroking Reported by Mothers Moderates the Effect of Prenatal Depression on Infant Behavioural and Physiological Outcomes. *PLoS ONE*, *7*(10), 45446. https://doi.org/10.1371/journal.pone.0045446

**Supplementary Table S1. Sensitivity analysis using teacher-reported attendance difficulties only (WCHADS)**

| **Predictor (Age 7)** | **Maternal depressive symptoms at age 9 (b, SE)** | **p** | **Attendance problems at age 9 (OR, 95% CI)** | **p** |
| --- | --- | --- | --- | --- |
| Maternal depressive symptoms | 0.54 (0.05) | <.001 | 0.99 (0.79–1.05) | .932 |
| Attendance problems | 0.26 (0.12) | .031 | 2.08 (1.07–4.07) | .032 |
| Deprivation | -0.09 (0.07) | .186 | 1.23 (0.75–2.04) | .414 |
| Maternal age at pregnancy | -0.02 (0.01) | .003 | 0.96 (0.91–1.00) | .090 |
| Degree or higher education | -0.15 (0.06) | .015 | 0.41 (0.21–0.81) | .010 |
| Child sex (female) | 0.04 (0.06) | .493 | 1.28 (0.77–2.12) | .339 |

**Note.** N = 707 (665 contributing to the maternal depression equation; 685 contributing to the attendance equation). Attendance difficulties were derived solely from teacher reports. Maternal depressive symptoms were standardised within wave.
